# Supplementary material for: Incident prolonged QT interval in midlife and late-life cognitive performance
Source: PLoS One. 2020 Feb 25;15(2):e0229519. doi: 10.1371/journal.pone.0229519 (PMC7041789; doi:10.1371/journal.pone.0229519)
Supplement: S1 Table — (DOCX) [file pone.0229519.s001.docx]

**SUPPLEMENTAL MATERIAL**

S1 Table. Variables included in the denominator of the inverse probability weight models.

|  | Incident QT  Exposure | Exam 2-3, no death | Exams 2-4, no death | Exams 2-4, no drop out | Exams 4-7, no death | Exams 4-7, no drop out |
| --- | --- | --- | --- | --- | --- | --- |
| Prolonged QT, Exam 2 *  Prolonged QT, Exam 3 * CASI |  |  |  |  | X | X |
| Months in study | X | X | X | X | X | X |
| Months^2^ | X | X | X | X | X | X |
|  |  |  |  |  |  |  |
| Non varying |  |  |  |  |  |  |
| Age, Exam 2 | X | X | X | X |  |  |
| Age, Exam 4 |  |  |  |  | X | X |
| Generation (Issei, Kibei, Nissei) | X | X | X | X | X | X |
| Education, Exam 1^a^ (years) |  | X | X | X | X | X |
| Clerical, sales, professional, or managerial job, Exam 1 ^a^ | X | X | X | X | X |  |
| Physical activity index, Exam 1 ^a^ | X | X | X | X |  | X |
| Chest depth, Exam 1 ^a^ (cm) | X | X | X | X |  |  |
| Alcohol use, Exam 1 ^a^ (grams) | X | X | X | X |  |  |
| Height, Exam 2 (meters) | X | X | X | X |  |  |
| Hypertension diagnosis, Exam 2 | X | X | X | X |  |  |
| Any *APOE ε4* allele |  |  |  |  | X | X |
| Alcohol use, Exam 4 (grams) |  |  |  |  | X |  |
| Hypertension diagnosis, Exam 4 |  |  |  |  | X |  |
| Hypocholesterolemic agent, Exam 4 |  |  |  |  | X |  |
| Height, Exam 4 (meters) |  |  |  |  | X |  |
| Coronary bypass history, Exam 4 |  |  |  |  | X |  |
| Dementia diagnosis, Exam 4 |  |  |  |  | X | X |
| Peripheral vascular disease, Exam 4 |  |  |  |  | X |  |
| Pulmonary disease, Exam 4 |  |  |  |  | X |  |
| Satisfaction with quality of life, Exam 4 |  |  |  |  | X |  |
| Group participation, Exam 4 |  |  |  |  |  | X |
|  |  |  |  |  |  |  |
| Time-dependent |  |  |  |  |  |  |
| Coronary heart disease or hypertensive heart disease diagnosis | X | X | X |  |  |  |
| Body mass index (kg/m^2^) | X | X | X | X | X | X |
| Systolic blood pressure | X | X | X | X |  | X |
| Diastolic blood pressure |  | X | X |  | X | X |
| Hypertension treatment | X | X | X | X |  | X |
| Cardiovascular disease-related prescription | X |  |  |  |  |  |
| Diabetes treatment | X |  |  |  |  |  |
| Current smoker |  | X | X |  | X | X |
| Cigarettes/day |  | X | X | X |  |  |
| Hypocholesterolemic agent |  | X | X | X |  |  |
| Stroke history |  | X | X |  | X | X |
| Myocardial infarction history |  |  |  |  |  |  |
| Married |  | X | X |  |  |  |
| Live with friends or family |  |  |  |  | X | X |
| Diabetes history |  |  |  |  |  |  |
| Self-rating general health (4-point scale) |  |  |  |  | X | X |
| Center for Epidemiologic Studies Depression scale |  |  |  |  | X | X |
| Physical activity index |  |  |  |  | X | X |
| Left and right grip strength |  |  |  |  | X |  |
| Exercise regularly |  |  |  |  | X |  |
| Activities of daily living score |  |  |  |  | X | X |
| Clinical Dementia Scale, sum of boxes |  |  |  |  |  | X |

1. Not asked at Exam 2, so Exam 1 responses were used.
