# Supplementary material for: Incident prolonged QT interval in midlife and late-life cognitive performance
Source: PLoS One. 2020 Feb 25;15(2):e0229519. doi: 10.1371/journal.pone.0229519 (PMC7041789; doi:10.1371/journal.pone.0229519)
Supplement: S2 Table — (DOCX) [file pone.0229519.s002.docx]

S2 Table. Visit-specific estimated inverse probability of attrition and inverse probability of exposure weights.

|  | Inverse Probability of Exposure Weights | | Inverse Probability of Death Weights | | Inverse Probability of Non-Death Drop-Out Weights | |
| --- | --- | --- | --- | --- | --- | --- |
| Exam (N) | Stabilized | Unstabilized | Stabilized | Unstabilized | Stabilized | Unstabilized |
| 2 (4737) | 1.00 | 1.00 | 1.00 | 1.00 | 1.00 | 1.00 |
| 3 (4582) | 1.00 | 1.96 | 1.00 | 1.01 | 1.00 | 1.02 |
| 4 (2511) | 0.99 | 2.29 | 1.00 | 1.53 | 1.00 | 1.18 |
| 5 (1877) | 0.99 | 2.26 | 1.00 | 1.14 | 1.00 | 1.14 |
| 6 (1397) | 0.99 | 2.24 | 1.01 | 1.17 | 1.03 | 1.13 |
| 7 (1060) | 0.99 | 2.27 | 0.97 | 1.15 | 1.00 | 1.14 |
