# Supplementary material for: Incident prolonged QT interval in midlife and late-life cognitive performance
Source: PLoS One. 2020 Feb 25;15(2):e0229519. doi: 10.1371/journal.pone.0229519 (PMC7041789; doi:10.1371/journal.pone.0229519)
Supplement: S3 Table — (DOCX) [file pone.0229519.s003.docx]

S3 Table. Mean of final non-truncated weights applied in estimation of the MSM at each visit with cognitive data

|  | Final Weights | |
| --- | --- | --- |
| Exam (N) | Mean of Stabilized Weights | Mean of Unstabilized Weights |
| 4 (2511) | 0.99 | 2.29 |
| 5 (1877) | 0.99 | 2.26 |
| 6 (1397) | 0.99 | 2.24 |
| 7 (1060) | 0.99 | 2.27 |
