# Supplementary material for: Incident prolonged QT interval in midlife and late-life cognitive performance
Source: PLoS One. 2020 Feb 25;15(2):e0229519. doi: 10.1371/journal.pone.0229519 (PMC7041789; doi:10.1371/journal.pone.0229519)
Supplement: S4 Table — (DOCX) [file pone.0229519.s004.docx]

S4 Table. Weighted demographics demonstrating inverse probability weights recover distribution of characteristics at baseline (Exam 2)

|  | **Exam 2**  **(n=4,737)** | **Exam 2 for the 2511 participants, unstabilized weighted** | **Exam 4**  **(n=2,511)** |
| --- | --- | --- | --- |
| Age at visit 2 (years) | 55.9 (5.4) | 56.0 (5.3) | 54.5 (4.5) |
| Generation |  |  |  |
| Issei | 472 (10%) | 9% | 149 (6%) |
| Kibei | 421 (9%) | 8% | 226 (9%) |
| Nisei | 3,844 (81%) | 83% | 2,136 (85%) |
| Education |  |  |  |
| None or primary | 1,052 (22%) | 22% | 449 (18%) |
| Intermediate | 1,292 (27%) | 27% | 682 (27%) |
| High School | 1,427 (30%) | 31% | 821 (33%) |
| Technical School | 475 (10%) | 10% | 278 (11%) |
| University | 471 (10%) | 10% | 281 (11%) |
| Clerical, sales, professional or managerial job | 1,384 (29%)* | 29% | 787 (31%) |
| Hypertension diagnosis | 568 (12%) | 15% | 225 (9%) |
| Alcohol (ounces/ month) (exam 1) | 13.3 (22.4)* | 13.4 (22.1) | 11.9 (20.3) |
| Height (cm) | 163 (6) | 163 (6) | 162 (6) |
| Chest depth (cm) | 19.3 (1.9)* | 19.3 (1.8) | 19.2 (1.8) |
| Physical Activity Index | 32.9 (4.6)* | 32.8 (4.4) | 32.9 (4.7) |
| Presence of at least one APOE-4 allele | -- |  | 462 (19%) |

*Evaluated at Exam 1
